# A comprehensive function analysis of LMO2 in different breast cancer subtypes

**DOI:** 10.18632/oncotarget.23542

**Published:** 2017-12-21

**Authors:** Ye Liu, Mei Yuan, Chao Wu, Tianhui Zhu, Wei Sun

**Affiliations:** ^1^ Reproductive Center, Tianjin First Center Hospital, Tianjin, China; ^2^ Department of Pharmacy, Beichen Hospital, Tianjin, China; ^3^ Laboratory of Molecular Genetics in School of Medicine, Nankai University, Tianjin, China

**Keywords:** LMO2, TCGA, breast cancer, PAM50 subtype

## Abstract

Breast cancer is the most common invasive cancer in women worldwide, and can be subdivided into Luminal A, Luminal B, Her2, and Basal subtype (the PAM50 subtyping system). The lmo2 gene was traditionally recognized as a proto-oncogene in hematopoietic system but its functions in breast cancers remained largely unclear. Based on the Cancer Genome Atlas (TCGA) breast cancer dataset, herein we found that the significantly LMO2-correlated genes in normal or malignant samples were enriched in rather divergent cellular pathways, suggesting the function complexity of LMO2 in breast tissues. Moreover, high LMO2 expression level was found to predict a shorter patient survival in Luminal A type whereas a better outcome in Her2 type. Correspondingly, LMO2 also revealed function diversities in different PAM50 subtypes. In Luminal A type, the LMO2 related genes were primarily enriched in cancer-promoting pathways, including VEGF production, stemness, PPAR signal pathways, MAPK cascade and cell cycle regulation. In Her2 type however, the LMO2 related genes lacked the enrichment on most of the generally cancer-related pathways and were particularly enriched in negative regulation of ErbB pathway as well as MAPK cascade, suggesting a potentially anti-oncogenic role of LMO2 on this subtype. Taken together, this study drew a comprehensive overview of divergent functions of LMO2 on breast cancers, provided additional evidence for the function complexity of LMO2 in solid tumors and suggested the potential usage of LMO2 as a PAM50 subtype dependent biomarker for breast cancer clinic in the precision medicine era.

## INTRODUCTION

The human *lmo2* gene was first cloned from an acute T lymphocytic leukemia (T-ALL) patient in 1990 [1]. Molecular function study of LMO2 revealed that it was widely expressed in a variety of tissue types [2, 3] and it located distinctively either in cytoplasm or in nucleus in different tissues [3]. As a nuclear transcriptional factor in hematopoietic-endothelial tissues, LMO2 primarily promoted embryonic hematopoiesis and angiogenesis [4–6], and specifically triggers T cell leukemia when ectopically expressed in T cell progenitors [7–9]. However, in most of the epithelial normal and malignant tissues, LMO2 primarily located in cytoplasm [3]. Till now several literatures have indicated complicated, even conflicting functions of LMO2 on tumor behaviors in different kinds of solid tumors [2, 10–12]. Notably, the LMO2 protein consists of only two tandem LIM domains which mediate protein-proteins interactions [13], and no matter in cytoplasm or nucleus, it always acted as a bridge or blocker molecule in a variety of protein complexes [14–18].

Breast cancer is a kind of highly heterogeneous disease with diversified biological and clinical characteristics. The PAM50 subtyping system, which can further subdivide all breast cancers into Luminal A, Luminal B, Her2, and Basal subtype based on their gene expression features or generally ER/PR and Her2 immuno-staining [19, 20], was widely employed on clinic. Our previous studies revealed that in breast cancers, LMO2 could attenuate the canonical Wnt-β-catenin signal pathway via binding with dishevelled-2 protein in a subtype-independent manner [2], while specifically in basal type breast cancer, LMO2 could promote tumor cell migration, invasion and metastasis via blocking the LIMK1-mediated cofilin1 phosphorylation [17]. In this study, primarily based on TCGA datasets, we further found that LMO2 expression level predicted patient survival inversely in Luminal A and Her2 subtype. Correspondingly, LMO2 was associated with rather different cellular functions and signal pathways in different breast cancer subtypes, as well as between normal and malignant breast tissues. These novel findings can help establishing a more comprehensive overview of the complicated functions of LMO2 in breast cancer.

## RESULTS

### LMO2 expression level differed in different breast cancer subtypes and indicated patient survival inversely in Luminal A and Her2 subtype

**Figure 1:**
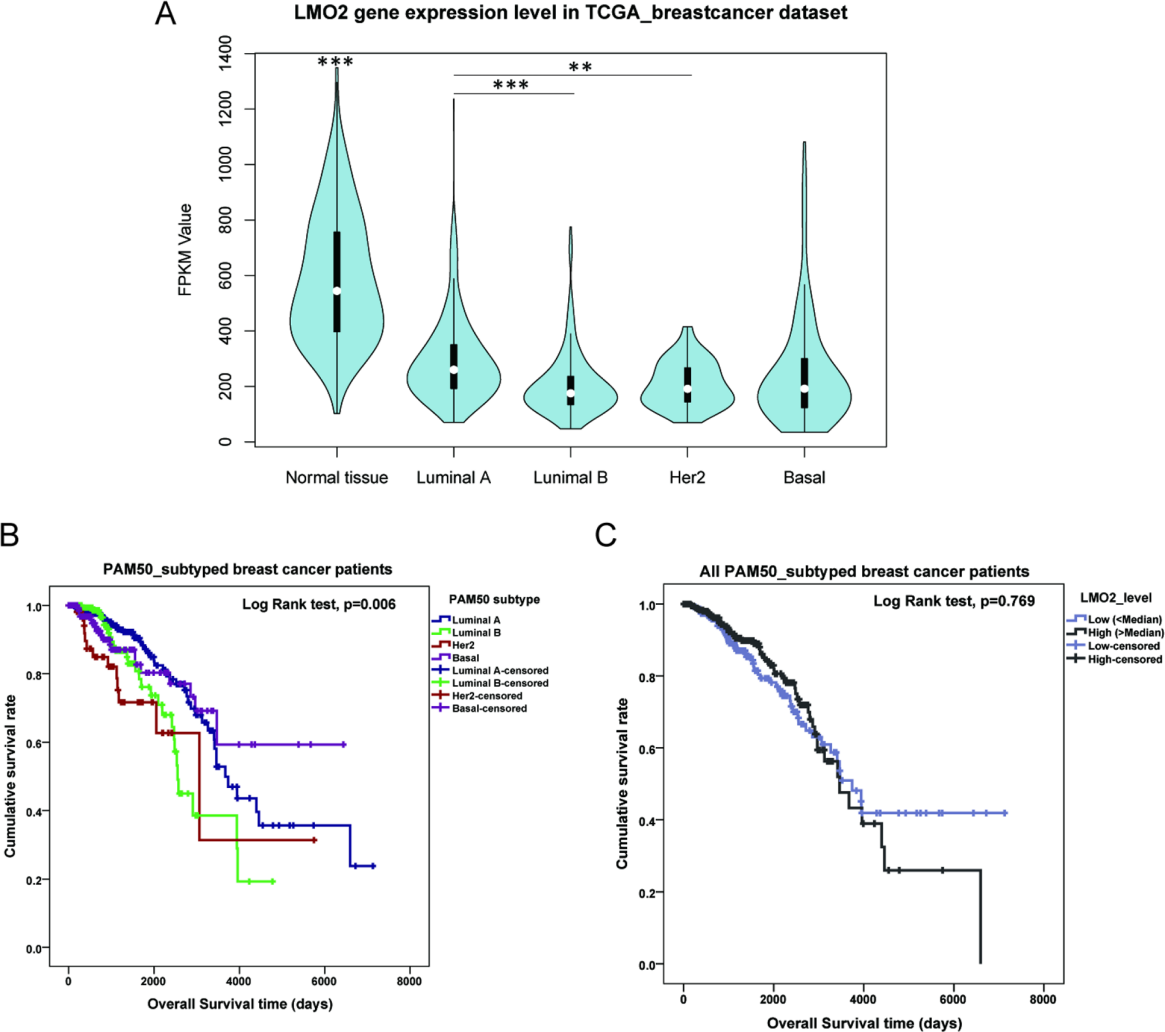

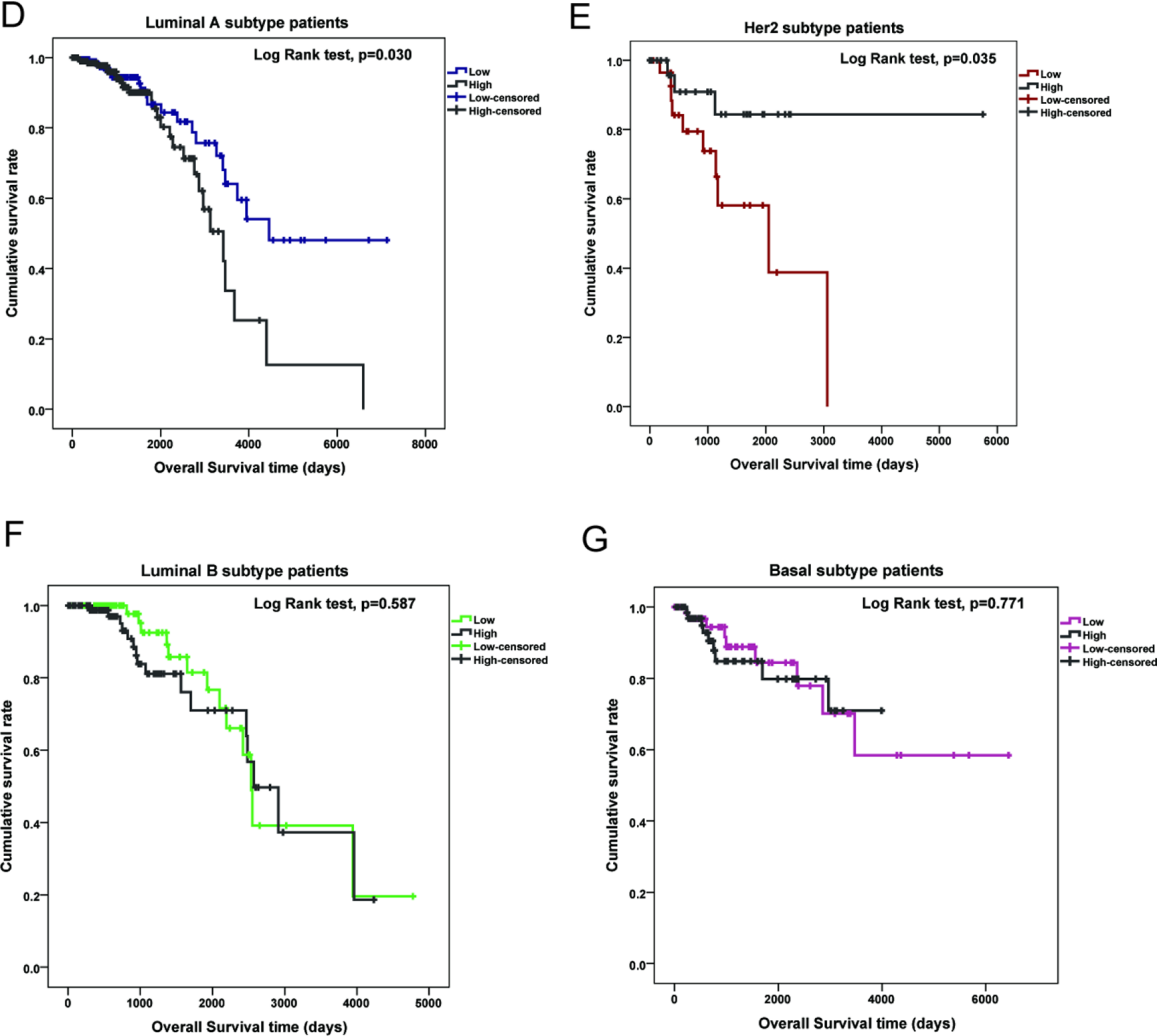
LMO2 expression profile and influence on patient survival in different PAM50 subtypes of breast cancer (**A**) Violin plot showing the medians and distribution of LMO2 mRNA expression level (FPKM value) in normal beast tissue samples and each PAM50 subtype of breast cancer samples from the TCGA breast invasive carcinoma RNA_seq dataset. Data were statistically analyzed by ANOVA and subsequently Tukey’s *t*-test, ^***^p < 0.001, ^**^p < 0.01. (**B**) Kaplan-Meier curves of patient survival of 4 PAM50 breast cancer subtypes. Survival data were obtained from the TCGA breast invasive carcinoma RNA_seq dataset. Log-Rank test *p*-values were shown in the plot. (**C**) Kaplan-Meier curves of patient survival of all breast cancer samples sub-grouped by LMO2 expression level. Patient samples were divided into high- and low-LMO2 expression groups based on the median expression value in the dataset. Log-Rank test *p*-values were shown in the plot. (**D**–**G**) Kaplan-Meier curves of breast cancer patient survival of each PAM50 subtype. Patient samples were divided into high- and low-LMO2 expression groups based on the median expression value in the dataset of each subtype. Log-Rank test *p*-values were shown in the plots.

Within the Cancer Genome Atlas (TCGA) breast invasive carcinoma RNA_seq dataset including 113 normal and 1095 primary malignant breast tissue samples, general statistical analysis revealed that the average LMO2 expression level (FPKM value) in normal tissues was significantly higher than any of the 4 subtypes of breast cancer (ANOVA followed by Tukey’s test, *p* < 0.001), while among the 4 subtypes, only a slight higher LMO2 expression was found in Luminal A type compared with Luminal B type (Tukey’s test, *p* < 0.001) or Her2 type (Tukey’s test, *p* = 0.002) (Figure 1A). Moreover, LMO2 has rather low mutation frequency in yet public datasets covered cancer types, and no LMO2 mutation has yet been detected in any breast cancer samples (Supplementary Figure 1A), implicating that LMO2 impacts tumor behaviors primarily via its expression fluctuation.

General survival analysis among different subtypes showed that the average patient survival in Luminal A and Basal group was longer than in Luminal B and Her2 group, indicating the latter two were the relatively poorer-prognostic subtypes in breast cancer (Figure 1B, Table 1, Supplementary Data Set Table 1). Notably, although LMO2 expression level had no prominent impact on patient survival in the whole sample group (Figure 1C), Luminal B group and Basal group (Figure 1F, 1G), high LMO2 expression indicated a shorter survival in Luminal A group whereas a longer survival and better prognosis in Her2 group (Log-Rank test, *p* < 0.05, Figure 1D, 1E, Table 1). These results suggested the probably diversified, subtype-dependent roles of LMO2 on breast cancer.

**Table 1:** Survival analysis of PAM50-subtyped breast cancer patient samples from TCGA

Category	Median survival time (days)	Mean survival time ± SD (days)	Log Rank test *p* value
**All samples**	3472	4154 ± 222	-
**LMO2_Low**	3738	4353 ± 302	*p* = 0.769
**LMO2_High**	3461	3822 ± 292	
**Luminal A**	3669	4308 ± 294	-
**LMO2_Low**	4456	4908 ± 393	*p* = 0.030 ^*^
**LMO2_High**	3418	3327 ± 355	
**Luminal B**	2551	2893 ± 221	-
**LMO2_Low**	2534	2936 ± 315	*p* = 0.587
**LMO2_High**	2573	2743 ± 261	
**Her2**	3036	3150 ± 676	-
**LMO2_Low**	2053	1897 ± 299	*p* = 0.035 ^*^
**LMO2_High**	-	4958 ± 425	
**Basal**	-	4655 ± 401	-
**LMO2_Low**	-	4687 ± 517	*p* = 0.771
**LMO2_High**	-	3269 ± 219	

^*^ Statistically significant

### The feature of LMO2 correlated gene profiles differed between normal and tumor breast tissues

**Figure 2:**
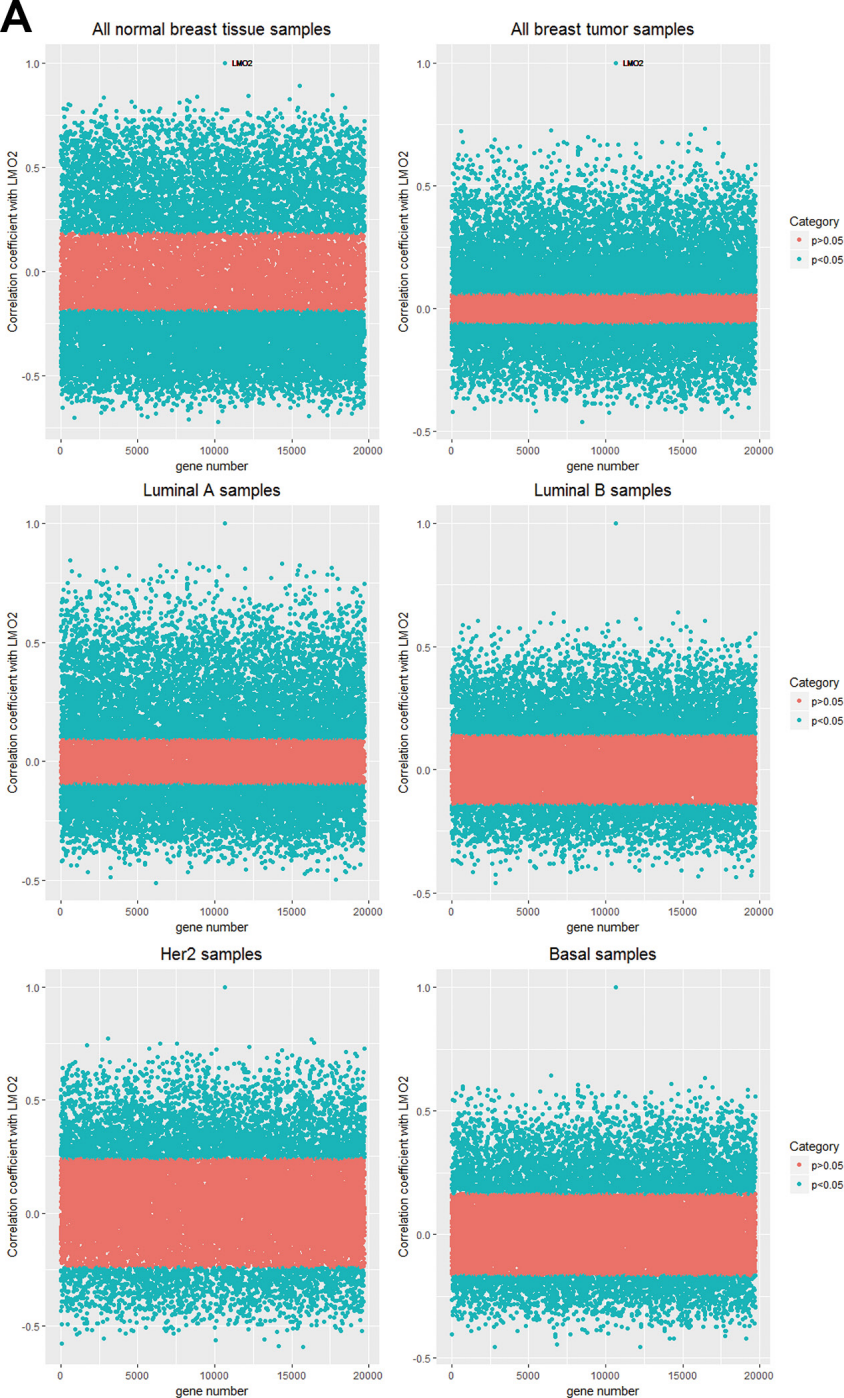

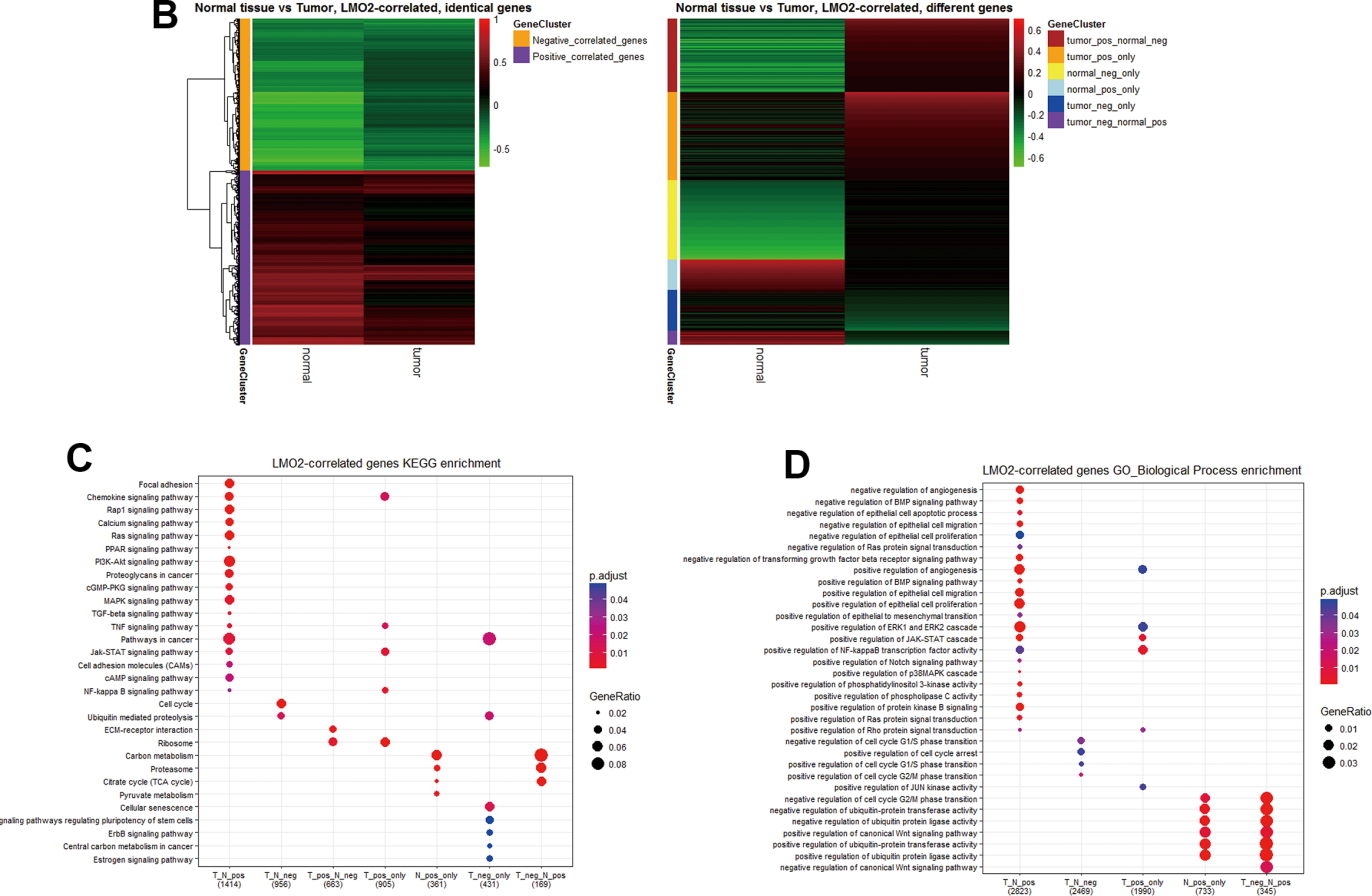

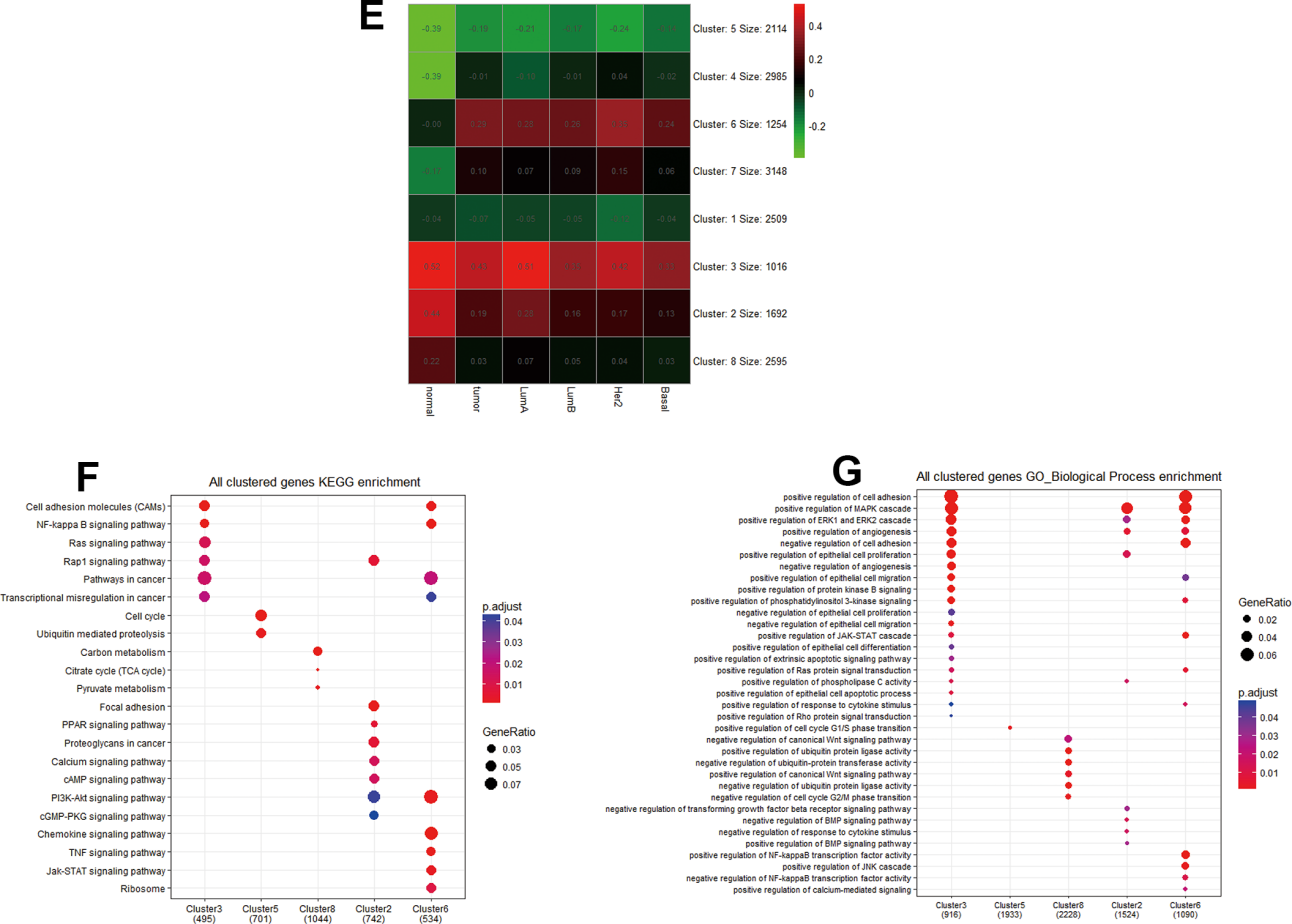
LMO2 function profiles in malignant and normal breast tissues (**A**) Dot plots showing the distribution of the correlation coefficient (*r*) of each gene to LMO2 calculated in different subgroups. The significantly correlated genes (*p* < 0.05) were marked in blue and non-significantly correlated genes (*p* ≥ 0.05) were marked in pink. The *p* = 0.05_*r*_cut-off values: *r*_(normal)_ = 0.185, *r*_(tumor)_ = 0.059, *r*_(LumA)_= 0.094, *r*_(LumB)_ = 0.141, *r*_(Her2)_ = 0.240, *r*_(Basal)_=0.164. (**B**) Heatmaps of identical and different LMO2-correlated genes in normal_vs_tumor subsets. All genes were clustered based on *r* values and *p*-values: tumor_vs_normal common positive (*r*_(tumor)_ > 0, *r*_(normal)_ > 0, *p*_(tumor)_< 0.05, *p*_(normal)_ < 0.05) or negative (*r*_(tumor)_< 0, *r*_(normal)_< 0, *p*_(tumor)_< 0.05, *p*_(normal)_ < 0.05) LMO2-correlated gene clusters; inversely tumor_vs_normal LMO2-correlated gene clusters (*r*_(tumor)_ > 0, *r*_(normal)_ < 0, *p*_(tumor)_ < 0.05, *p*_(normal)_ < 0.05; or inversely *r*_(tumor)_ < 0, *r*_(normal)_ > 0, *p*_(tumor)_ < 0.05, *p*_(normal)_ < 0.05); tumor-specific positive (*r*_(tumor)_ > 0, *p*_(tumor)_ < 0.05, *p*_(normal)_ ≥ 0.05) or negative (*r*_(tumor)_ < 0, *p*_(tumor)_ < 0.05, *p*_(normal)_ ≥ 0.05) LMO2-correlated gene clusters; and normal tissue-specific positive (*r*_(normal)_ > 0, *p*_(normal)_ < 0.05, *p*_(tumor)_ ≥ 0.05) or negative (*r*_(normal)_ < 0, *p*_(normal)_ < 0.05, *p*_(tumor)_ ≥ 0.05) LMO2-correlated gene clusters. Relative information was marked on the plots. (**C**, **D**) Dot plots showing the selected cancer-related terms of KEGG or additional GO-Biological Process (GO_BP) enrichment assay on each gene cluster grouped in B. Relative information was marked on the plots. (**E**) Heatmap of unsupervised, 8 means clustering on all genes based on the LMO2-correlation coefficient (*r*) in different categories. Cluster2, normal-high-tumor-low-positive LMO2-correlated geneset; Cluster3, common positive LMO2-correlated geneset; Cluster4, normal negative LMO2-correlated geneset; Cluster5, common negative LMO2-correlated geneset; Cluster6, tumor positive LMO2-correlated geneset; Cluster8, normal positive LMO2-correlated geneset; Cluster1, 7, non-significantly correlated genesets. The means of *r*-values of each cell and other relative information were marked on the plot. (**F**, **G**) Dot plots showing the selected cancer-related terms of KEGG or additional GO-Biological Process (GO_BP) enrichment assay on each gene cluster grouped in E. Relative information was marked on the plots.

As the enrichment of LMO2-correlated genes in each group (Figure 2A, Supplementary Data Set Table 2.1) can predict the primarily functional aspects of LMO2 in that certain type, all LMO2-correlated genes except LMO2 itself (*r* = 1) in normal and tumor group were firstly clustered based on *r* values (Figure 2B) and following KEGG and GO-Biological Process enrichment assays were performed on all these clusters (Supplementary Figure 2A, 2B, Supplementary Data Set Table 2.2, 2.3). For those most properly cancer-related terms, KEGG analysis revealed that the tumor-normal common positive or negative LMO2-correlated genes were enriched in some classical pathways, such as cell cycle, Ras-MAPK pathway, PI3K-AKT pathway, JAK-STAT pathway, TGF-beta signal pathway, TNF signal pathway and pathways involved in regulating cytoskeleton and cell adhesion, indicating the primary indistinctive functions of LMO2 in breast tissues. Remarkably, tumor-specific positive LMO2-correlated genes were enriched in Ribosome and Extracellular matrix (ECM) receptor interaction while negative LMO2-correlated genes were specifically enriched in some tightly cancer-related pathways, including cellular senescence, stemness regulating pathway, ErbB (Her2) pathway, Estrogen pathway and central carbon metabolism in cancer. In contrast, normal-specific positive LMO2-correlated genes were enriched in some metabolism pathways potentially related to cancer metabolism, including carbon metabolism, Citrate cycle (TCA cycle) and pyruvate metabolism (Figure 2C). Interestingly in the additional GO-Biological Process (BP) analysis, many enriched cellular processes and signal pathways revealed dual-directional regulation features, such as both positive and negative regulation of angiogenesis, epithelial cell migration, proliferation, TGF-beta/BMP signal pathway, Ras-MAPK pathway for the tumor-normal common positive gene cluster; both positive and negative regulation of cell cycle phase transition for the tumor-normal common negative gene cluster and both positive and negative regulation of protein ubiquitination process, canonical Wnt signal pathway for normal-specific positive gene cluster. These terms might represent the uncertain or further condition-dependent effect of LMO2. Notably, the tumor-normal common positive LMO2-correlated genes were also enriched in the negative regulation of apoptosis and positive regulation of EMT, JAK-STAT, PI3K-AKT, Notch and cytoskeleton remodeling (Rho protein) pathway, while the normal-specific positive LMO2-correlated genes were particularly enriched in the negative regulation of cell cycle G2/M phase transition (Figure 2D).

Alternatively, we unsupervised clustered all genes except LMO2 into 8 clusters based on *r*-values in different groups (Figure 2E). The following KEGG enrichment analysis (Supplementary Figure 2C, Supplementary Data Set Table 2.4) revealed that similarly with previous, the common-positive genes (Cluster2, 3) were enriched in Ras-MAPK pathway, PI3K-AKT pathway, general cancer pathways, pathways involved in regulating cytoskeleton and cell adhesion, and the common-negative genes (Cluster5) were enriched in cell cycle, respectively; the tumor-specific positive genes (Cluster6) were enriched in Chemokine pathway, JAK-STAT pathway, TNF signal pathway, Ribosome; and the normal-specific positive genes (Cluster8) were enriched in metabolism pathways including carbon metabolism, Citrate cycle (TCA cycle) and pyruvate metabolism (Figure 2F). GO-BP analysis (Supplementary Figure 2D, Supplementary Data Set Table 2.5) revealed almost identical result with previous as well (Figure 2G).

In summary, these results suggested that LMO2 was positively associated with some metabolism pathways in normal breast tissue, negatively associated with some cancer-related pathways, such as stemness regulation, ErbB pathway, Estrogen pathway and central carbon metabolism in malignant breast tissue, and associated with many dual-directional functions in both normal and malignant breast tissues.

### The feature of LMO2 correlated gene profiles in breast cancer samples differed among different PAM50 subtypes

**Figure 3:**
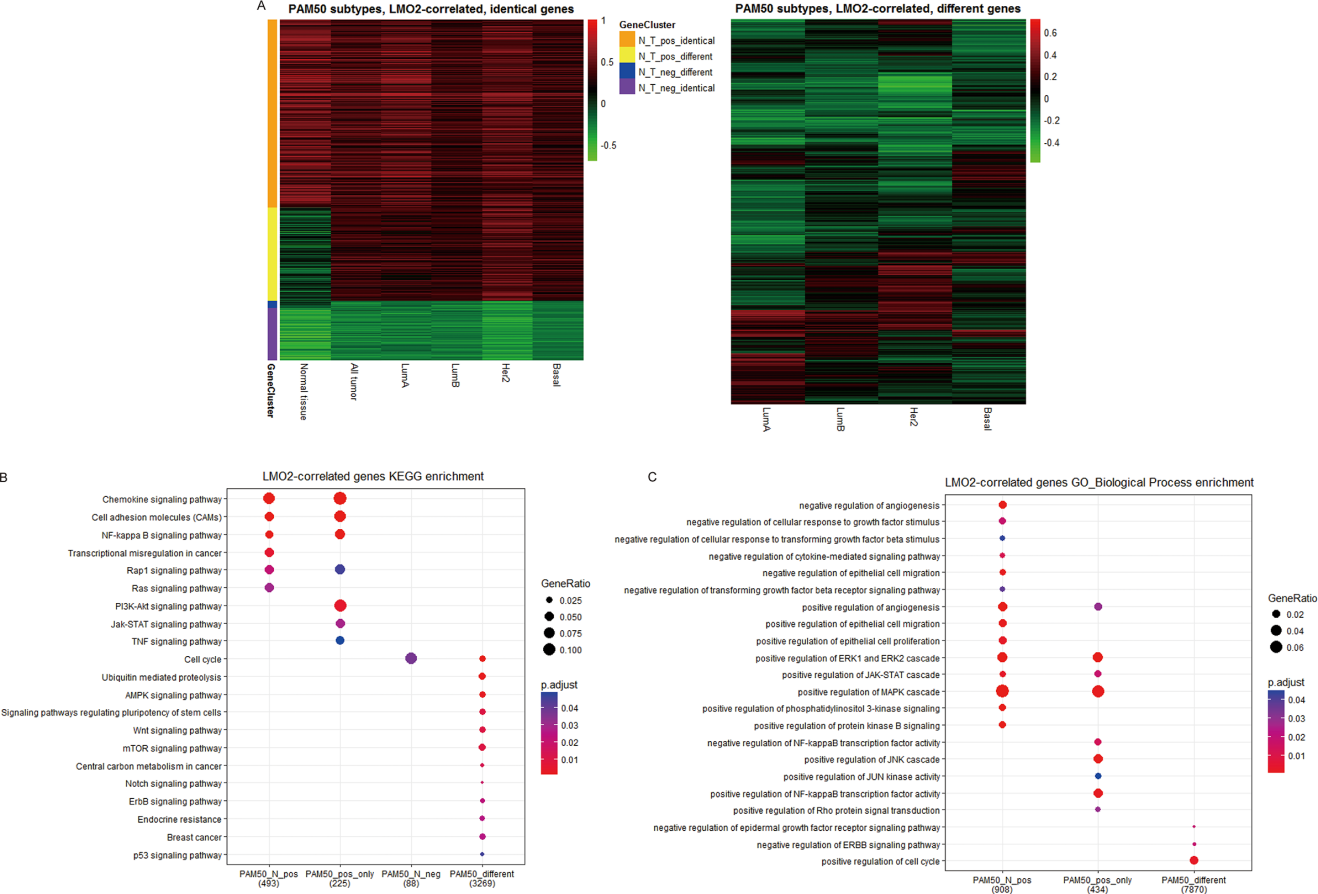

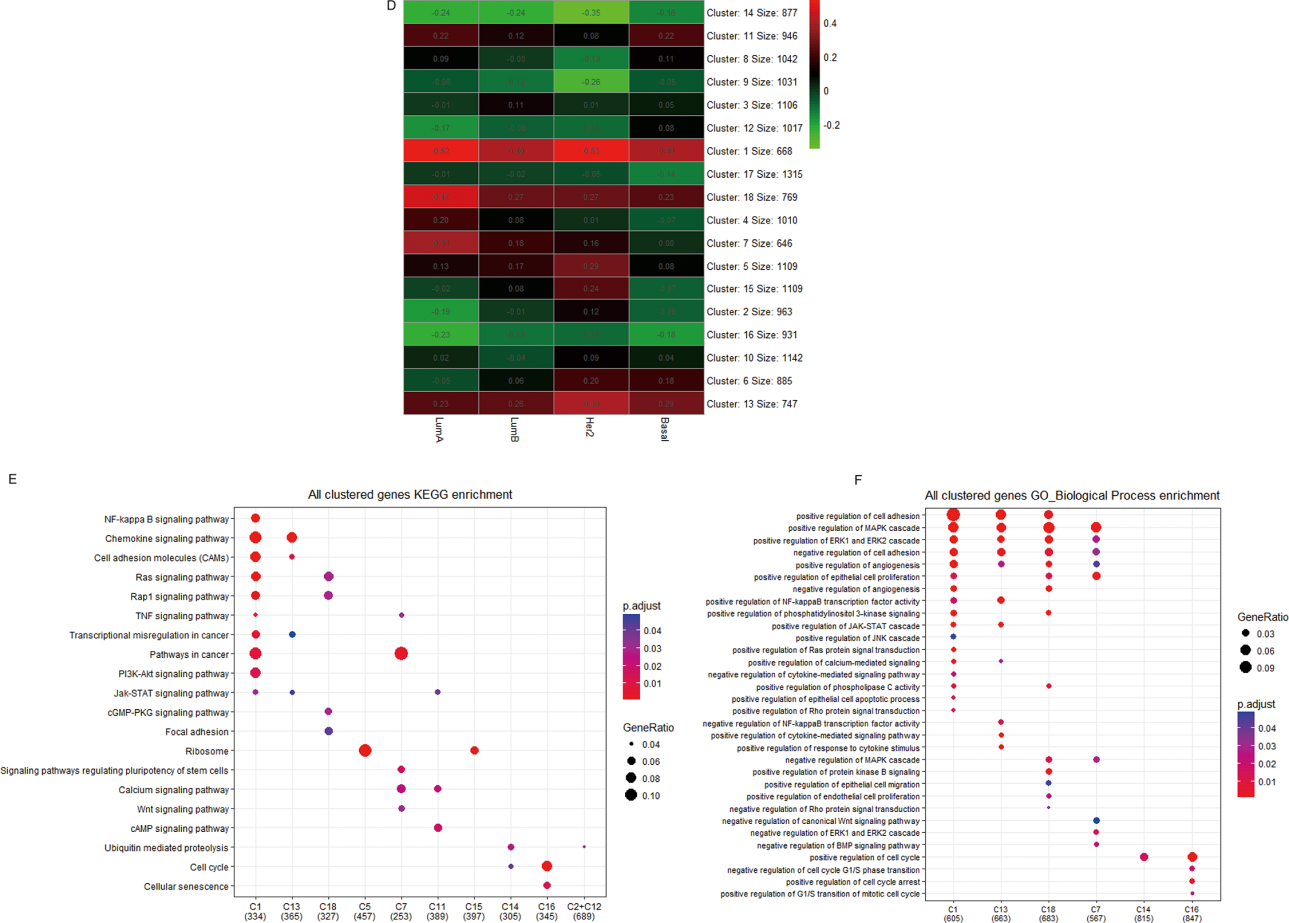

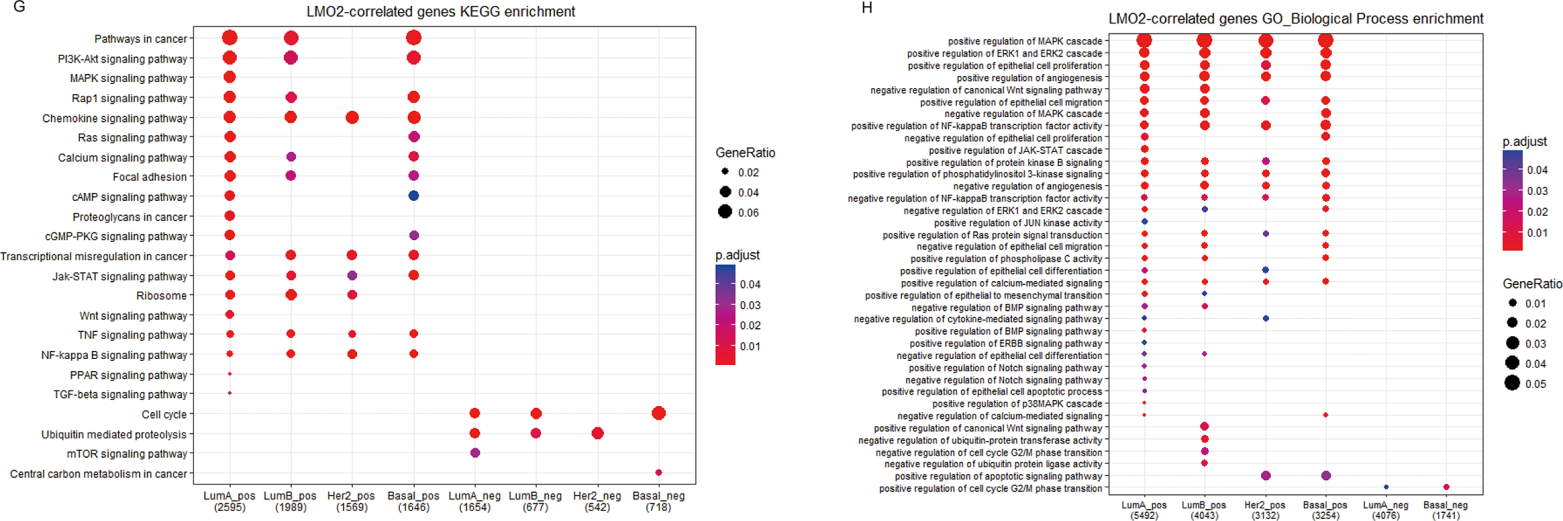
LMO2 function profiles in different PAM50 subtypes of breast cancer samples (**A**) Heatmaps of identical and different LMO2-correlated genes among each PAM50 subtype. PAM50 subtype identical gene clusters: all subtypes *p* < 0.05; PAM50 subtype different gene cluster: at least *p* < 0.05 in one subtype while *p* > 0.05 in another. Other relative information was marked on the plots. (**B**, **C**) Dot plots showing the selected cancer-related terms of KEGG or additional GO-Biological Process (GO_BP) enrichment assay on each gene cluster grouped in A. Relative information was marked on the plots. (**D**) Heatmap of unsupervised, 18 means clustering on all genes based on the LMO2-correlation coefficient (*r*) in different PAM50 subtype categories: Cluster1, all-strong-positive geneset; Cluster13, all-less-strong-positive geneset; Cluster18, all-positive while LumA-specific-stronger geneset; Cluster5, LumA+LumB+Her2-positive geneset; Cluster7, LumA+LumB-positive geneset; Cluster11, LumA+Basal-positive geneset; Cluster4, LumA-positive geneset; Cluster15, Her2-positive geneset; Cluster6, Basal-positive geneset; Cluster14, all-negative geneset; Cluster9, Her2-negative geneset; Cluster2+12, LumA-negative geneset; Cluster16, LumA+Basal negative geneset; Cluster3, 8, 10, 17, non-significantly correlated genesets. The means of *r*-values of each cell and other relative information were marked on the plot. (**E**, **F**) Dot plots showing the selected cancer-related terms of KEGG or additional GO-Biological Process (GO_BP) enrichment assay on each gene cluster grouped in D. Relative information was marked on the plots. (**G**, **H**) Dot plots showing the selected cancer-related terms of KEGG or additional GO-Biological Process (GO_BP) enrichment assay on the clusters of significantly LMO2-correlated genes in each PAM50 subgroup independently. Relative information was marked on the plots.

To further investigate the potentially function diversity of LMO2, we divided all LMO2-correlated genes (except LMO2 itself) into PAM50 subtype identical group (*p* < 0.05 in all of the 4 subtypes) or different group (the rests), and the PAM50 identical group was further clustered into positive LMO2-correlated, identical or different with normal tissue, and negative LMO2-correlated, identical or different with normal tissue clusters (Figure 3A). KEGG analysis on these gene clusters (Supplementary Figure 3A, Supplementary Data Set Table 3.1) showed that the PAM50 subtype identical genes revealed reasonably almost identical enrichment profiles with previous analysis on all tumor and normal samples. Notably, the PAM50 subtype different genes were particularly enriched in cell cycle, ubiquitination regulation, AMPK-mTOR, Wnt, Notch, ErbB, stemness and p53 pathways, central carbon metabolism and endocrine resistant (Figure 3B), implementing that LMO2 might play different roles on different subtypes via these pathways. In addition, GO-BP analysis (Supplementary Figure 3B, Supplementary Data Set Table 3.2) revealed that the PAM50 subtype different genes were further enriched in negative regulation of EGFR signal pathway and positive regulation of cell cycle (Figure 3C).

Also alternatively, we unsupervised clustered all genes except LMO2 into 18 clusters based on *r*-values in different PAM50 subgroups (Figure 3D). In KEGG analysis (Supplementary Figure 3C, Supplementary Data Set Table 3.3), we noticed that the enriched terms from the common positive or negative clusters (Cluster1, 13, 18 and 4) were nearly identical with previous. Remarkably, positive LMO2-correlated genes in Basal group lacked the enrichment of Ribosome (Cluster5); in Her2 group were particularly enriched in Ribosome (Cluster15); in common Luminal group (including both Luminal A and Luminal B, Cluster7) were enriched in stemness and Wnt pathways; in both Luminal A and Basal group (Cluster11) were enriched in cAMP signal pathway; meanwhile, the negative LMO2-correlated genes were particularly enriched in cell senescence in common Luminal A and Basal subtypes (Cluster 16) (Figure 3E). Additional GO-BP analysis (Supplementary Figure 3D, Supplementary Data Set Table 3.4) revealed that the positive LMO2-correlated genes in common Luminal group (Cluster7) were enriched in negative regulation of canonical Wnt and BMP signal pathways, while the negative LMO2-correlated genes in all subtypes (Cluster14) were enriched in positive regulation of cell cycle (Figure 3F).

Finally, the KEGG and GO-BP analysis on the LMO2-correlated genesets of each PAM50 subtype independently (Supplementary Figure 3E, F; Supplementary Data Set Tables 3.5, 3.6) revealed that in Luminal A type, positive LMO2-correlated genes were specifically enriched in PPAR, TGF-beta pathways and positive regulation of ErbB pathway, negative LMO2-correlated genes were enriched in mTOR pathway; in Luminal B type, LMO2-positive correlated genes were specifically enriched in negative regulation of ubiquintination and G2/M phase transition while lacked the enrichment of positive regulation of apoptosis compared with the other 3 subtypes; in Basal type, positive LMO2-correlated genes lacked the enrichment of Ribosome, negative LMO2-correlated genes were specifically enriched in central carbon metabolism in cancer while lacked the enrichment of ubiquitination regulation compared with the other 3 subtypes; interestingly in Her2 type, the LMO2-correlated genes lacked the enrichment in many pathways compared with the other 3 subtypes, including the general pathways in cancer and cell cycle regulation; moreover, the positive LMO2-correlated genes in common Luminal type (Luminal A + Luminal B) were also enriched in the positive regulation of epithelial to mesenchymal transition (EMT) (Figure 3G, 3H).

Taken together, these analyses indicated the diversified, multi-directionally LMO2 preferred functions in different PAM50 subtypes of breast cancer. In summary, LMO2 tended to correlate to oncogenic pathways in Luminal types, such as regulation of stemness and EMT, and particularly correlated to PPAR, TGF-beta/BMP and mTOR pathways in Luminal A type. In Basal type, LMO2 primarily negatively correlated to central carbon metabolism in cancer but also cell senescence. In Her2 type however, LMO2 lacked the correlation with most of the cancer-related pathways.

### LMO2 exhibited rather different effects on Luminal A type and Her2 type breast cancers

**Figure 4:**
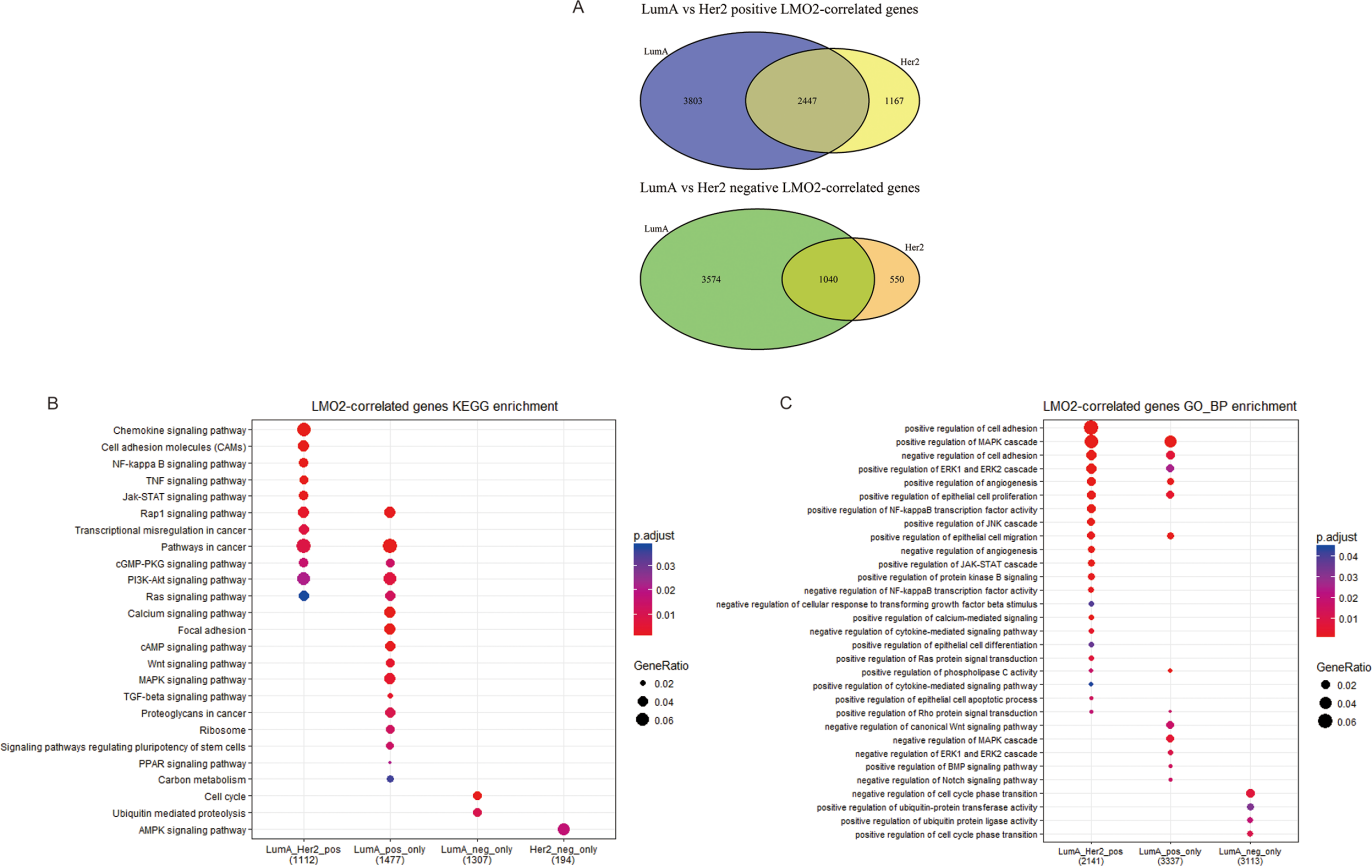

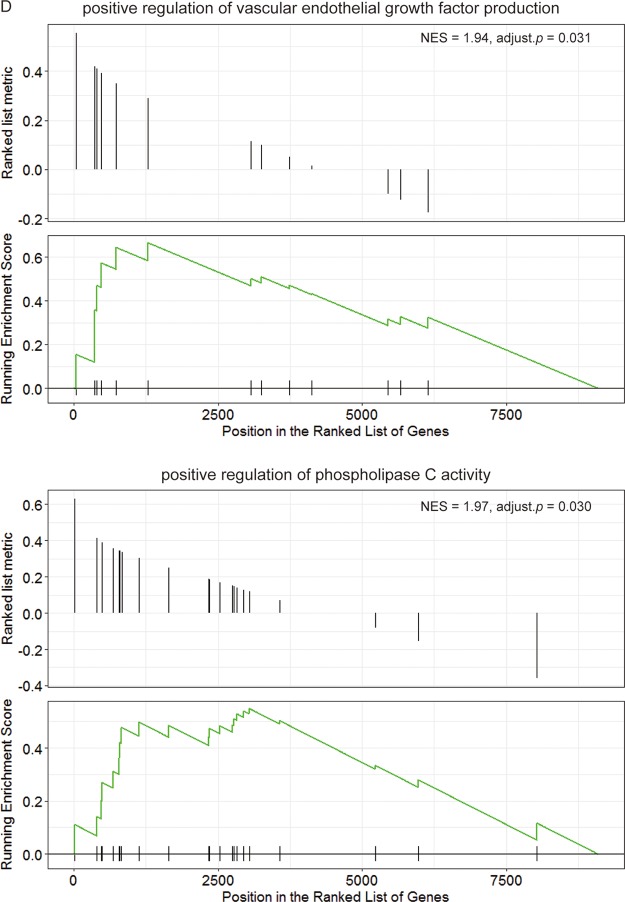

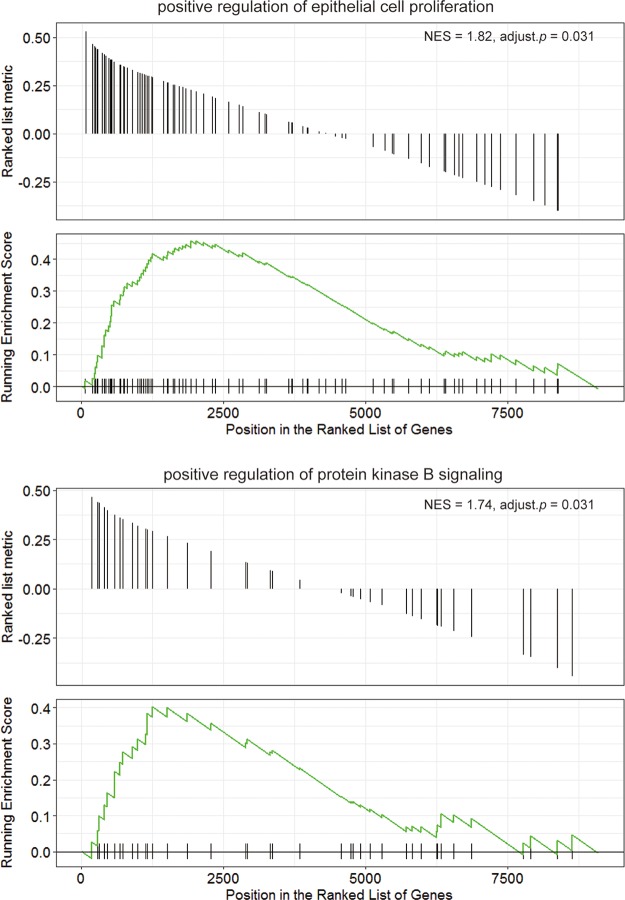

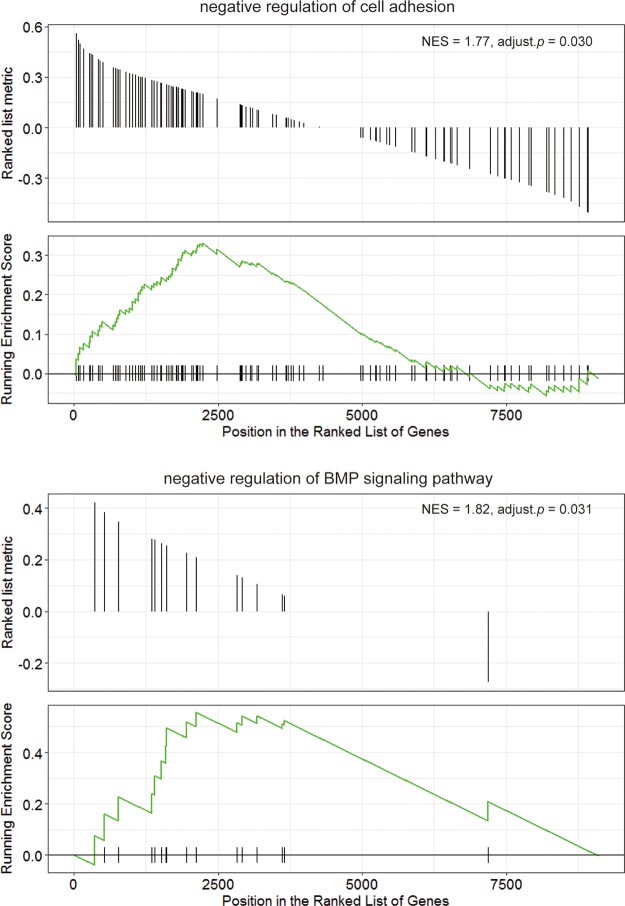
Functional difference of LMO2 between Luminal A and Her2 subtype of breast cancers in TCGA dataset (**A**) Venn map showing the intersected and unique fractions of LMO2-correlated genes in Luminal A and Her2 subgroups. Relative Gene numbers were marked on the plots. (**B**, **C**) Dot plots showing the selected cancer-related terms of KEGG or additional GO-Biological Process (GO_BP) enrichment assay on each geneset grouped in A. Relative information was marked on the plots. (**D**) GSEA plots of selected, significantly enriched cancer-associated terms (adjust. *p* value < 0.05) in the dataset of all LumA-Her2 differently LMO2-correlated genes (the 4 unique sections in A) ranked by (*r*_(LumA)_-*r*_(Her2)_) value. ES values, adjust. *p* valuse and other relative information were marked on the plots.

Previous data showed that LMO2 had totally inverse impact on patient survival and rather different function associations between Luminal A type and Her2 type. Herein we further divided the LMO2-correlated genes in these two groups into the common-LumA-Her2 positive/negative, LumA-specific positive/negative and Her2-specific positive/negative groups (Figure 4A). The following KEGG and GO-BP analysis (Supplementary Figure 4A, 4B; Supplementary Data Set Tables 4.1, 4.2) revealed that consistent with previous, LumA-specific positive/negative LMO2-correlated genes were enriched in stemness, PPAR signal pathways and cell cycle, while notably, Her2-specific negative LMO2-correlated genes were specifically enriched in AMPK pathway, which functioned on the opposite direction of mTOR pathway that enriched by Luminal A negative genes in previous (Figure 4B, 4C). In addition, in GSEA analysis (Supplementary Data Set Tables 4.3, 4.4), although some results showed dual-directional function enrichment, such as both positive and negative regulation of epithelial cell migration, angiogenesis and MAPK pathway (Supplementary Figure 4C), the (*r*_(LumA)_-*r*_(Her2)_) value ranked genelist specifically hit on the terms of positive regulation on VEGF production, epithelial cell proliferation, phospholipase C (PLC) activity, PI3K-AKT (PKB) pathway, negative regulation on cell adhesion and BMP signal pathway (Figure 4D). These results further supported the intensely oncogenic-preferred functions of LMO2 in Luminal A type compared to Her2 type in breast cancers.

**Figure 5:**
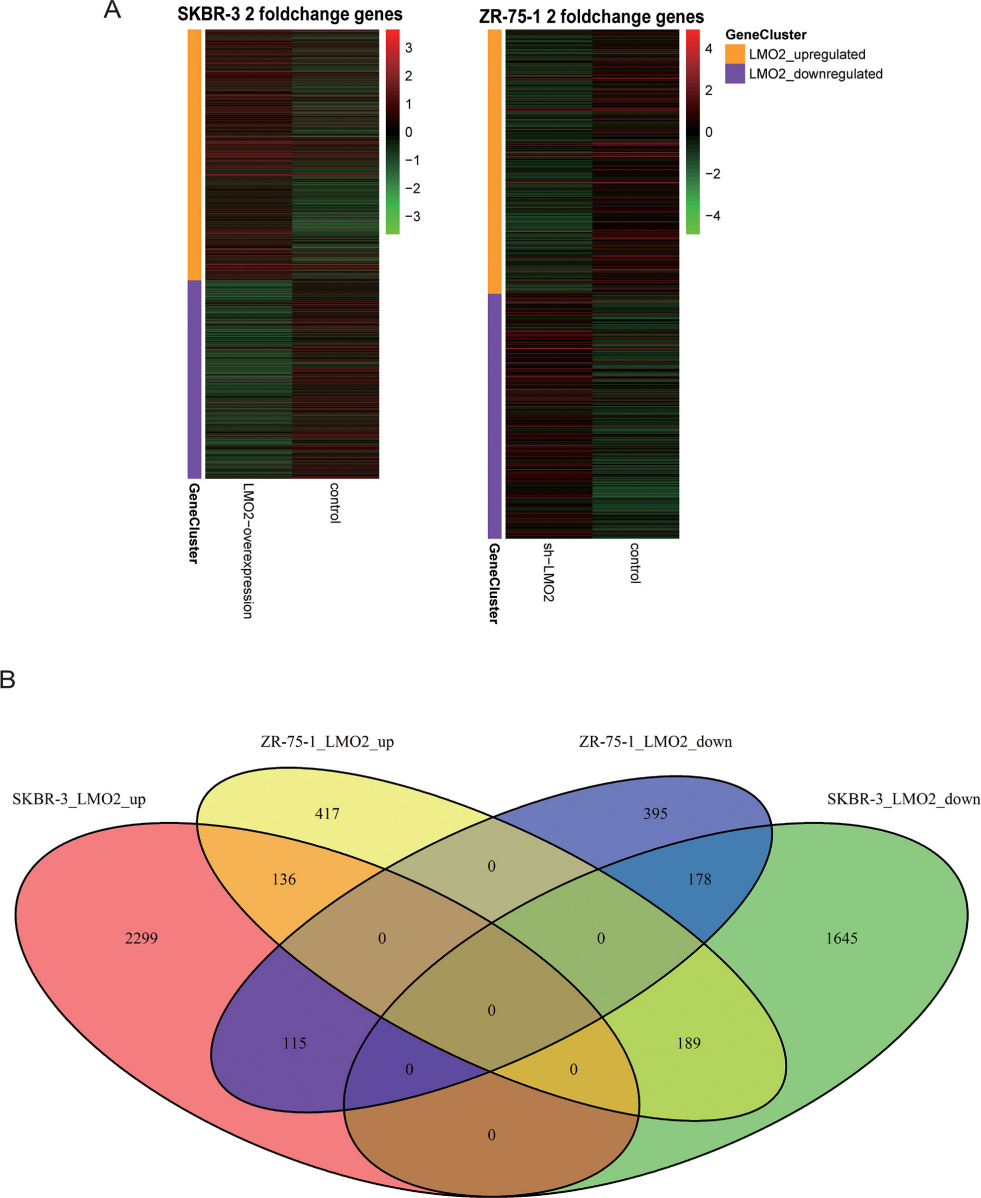

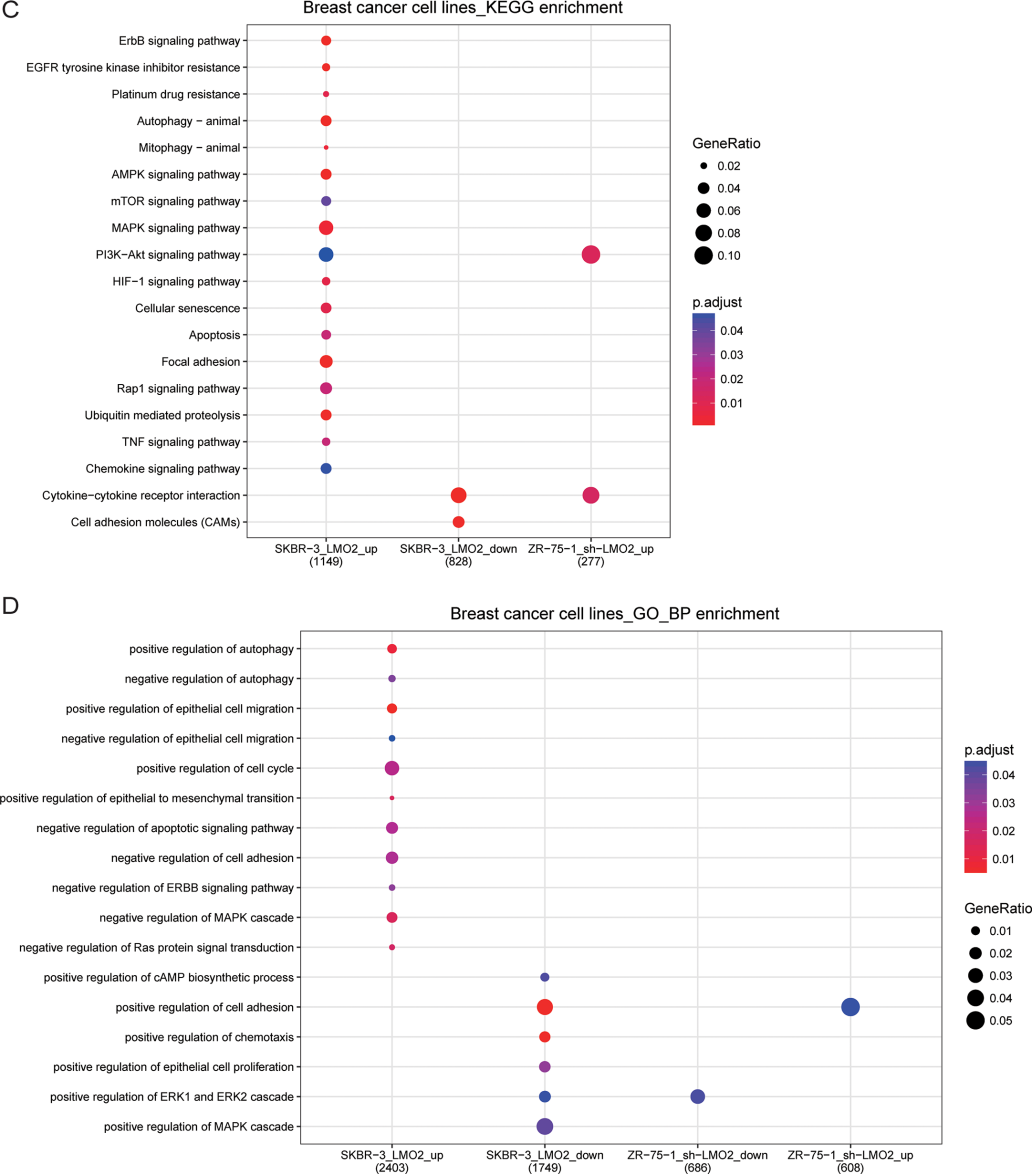
Functional difference of LMO2 in Luminal A and Her2 like breast cancer cell lines (**A**) Heatmaps of all ≥2 fold change genes in LMO2-modified SKBR-3 and ZR-75-1 cell strains compared with their relevant control cells. (**B**) Venn map showing the intersected parts of common LMO2_upregulated genes, common LMO2_downregulated genes and inversely LMO2-regulated genes in SKBR-3 and ZR-75-1 cells. (**C**, **D**) Dot plots showing the selected cancer-related terms of KEGG or additional GO-Biological Process (GO_BP) enrichment assay on each ≥ 2 fold change geneset of SKBR-3 and ZR-75-1 cells. Relative information was marked on the plots.

Moreover, in microarray data of LMO2 knocking-down/control ZR-75-1 cell strains (Luminal A like) and LMO2-overexpression/control SKBR-3 cell strains (Her2 like), ≥ 2 fold changed genes in each treatment/control were selected (Figure 5A). Among all these genes, there were 136 common LMO2_upregulated genes and 178 common LMO2_downregulated genes, while 115 genes upregulated in SKBR-3 cell but downregulated in ZR-75-1 cell and 189 genes regulated in the opposite manner, as the LMO2 inversely regulated genes in these two cell lines (Figure 5B). KEGG and GO_BP analysis on this two cell lines independently (Supplementary Figure 5A, B, Supplementary Data Set Tables 5.1, 5.2) revealed that in SKBR-3 cell, LMO2 functions were enriched in dual-directional regulation of autophage and cell migration, in positive regulation of cell cycle and EMT, and in negative regulation of cell apoptosis, adhesion, chemotaxis and most pivotally, cell proliferation and the ERBB-Ras-MAPK-ERK signal pathway. In contrast, in ZR-75-1 cell, LMO2 functions were enriched in positive regulation of ERK cascade and negative regulation of cell adhesion. In both of the two cells, LMO2 functions were associated with PI3K-AKT pathway and cytokine-cytokine receptor interaction (Figure 5C, 5D). In addition, KEGG and GO_BP analysis were further performed on the 4 intersected genesets in Figure 5B, however, few cancer-related terms were hit except cytokine-cytokine receptor interaction which was in accordance with the result in Figure 5C (Supplementary Data Set Tables 5.3, 5.4). These results further supported the issue of much LMO2 function difference between Luminal A and Her2 type breast cancers and critically, indicated the negative regulation on the ERBB signal pathway by LMO2 in Her2 type breast cancer cells.

## DISCUSSION

Although traditionally recognized as a pivotal transcriptional regulator in hematopoietic and vascular endothelial systems, more and more evidence showed that LMO2 played rather complicated functions in multiple solid tumors as well, particularly with specifically cytoplamic location [2, 3]. Till now some published literatures indicated that LMO2 expression was increased in low grade glioblastoma, whereas decreased in head and neck, lung, colorectal, breast, renal, uterine corpus endometrioid, and cervical carcinomas [2], meanwhile, LMO2 played oncogenic role in glioblastoma [10] and prostate carcinoma [11], but was a good prognostic marker for diffuse large B cell lymphoma (DLBCL) [21–23], acute B lymphocytic leukemia (B-ALL) [24] and pancreatic carcinoma [12]. Till now there still lacks systematic reports on LMO2 functions in breast cancer, in some of our preliminary studies [2, 17], we had demonstrated that LMO2 could attenuate the canonical Wnt-β-catenin pathway via binding with dishevelled-2 protein in a subtype-independent manner and specifically played a function of promoting tumor cell migration, invasion and metastasis via blocking on LIMK1-mediated phosphorylation of cofilin1 in basal type breast cancer. In this study, we further found that LMO2 were associated with different cellular functions between malignant and normal breast tissues, as well as among different PAM50 subtypes. Remarkably, in Luminal A type, LMO2 related genes were specifically enriched in VEGF production, stemness, PPAR signal pathways and cell cycle regulation, indicating rationally oncogenic functions; whereas in Her2 type, the LMO2 related genes lacked the enrichment on many of the cancer-related pathways but particularly enriched in the negative regulation of ERBB-Ras-MAPK-ERK signal pathway, suggesting a largely tumor-suppressive effect of LMO2. These results further supported the fact of functional complexity of LMO2 in different kinds of solid tumors.

As a member of LIM domain superfamily protein and consisting of only two tandem LIM domains, LMO2 revealed the unique molecular pattern that mediated protein-protein interaction as either an adaptor or blocker molecule [2, 13, 17, 18]. Following this issue, we tended to explain the functional complexity of LMO2 in such model: the structure feature of LMO2 allows it to bind to diverse partners from multiple cellular pathways simultaneously, and the predominant function of LMO2 in a certain cell type depends on not only the abundance of LMO2 itself but also the abundance of LMO2 preferred partners, which can consume LMO2 molecules competitively and guide LMO2 to different function pathways (Supplementary Figure 1B).

Different subtypes of breast cancers have different preferred clinical therapeutic strategies, such as anti-Estrogen therapy for Luminal A/B type, ERBB2 (Her2) target therapy for Her2/Luminal B type and chemotherapy for Basal type [19]. Herein we found that in general Luminal A type breast cancer samples had relatively higher LMO2 expression level than other 3 types in initial-diagnosed tumors and no literatures about the LMO2 expression level variation in tumor samples upon relative therapies have been reported. Particularly, LMO2 had totally inverse impact on patient survival in Luminal A and Her2 type. High LMO2 expression indicated a shorter survival in Luminal A type whereas a longer survival and better prognosis in Her2 type. Correspondingly, LMO2 exhibited primarily oncogenic features in Luminal A type while tumor-suppressive functions in Her2 type. All such features of LMO2 suggested that it was quite suitable to be developed as prognostic marker on clinic for predicting patient survival discriminatively in certain breast cancer subtype.

Taken together, our study drew a comprehensive overview of divergent functions of LMO2 on breast cancers. These results provide additional evidence for the function complexity of LMO2 in solid tumors and suggest the potential usage of LMO2 as a PAM50 subtype dependent biomarker for breast cancer clinic in the future.

## MATERIALS AND METHODS

### Online datasets and data processing

The TCGA breast invasive carcinoma RNA_seq dataset (Level 3 data) was downloaded from the UCSC Cancer Genomics Browser (https://genome-cancer.ucsc.edu/). The LMO2 expression data matrix and clinical information file were matched by sample ID for each sample (Supplementary Data Set Table 6). Statistical analysis of LMO2 expression was performed with IBM SPSS Statistics version 20.0 (SPSS Inc., Chicago, IL). The correlation coefficient (*r* value) and relevant *p*-value of each gene to LMO2 in different subgroups were calculated by R. All *p* < 0.05 genes were marked as significantly LMO2-correlated genes. The *r* cut-off values for *p* = 0.05 in normal, all tumor, Luminal A, Luminal B, Her2 and Basal subsets were 0.185, 0.059, 0.094, 0.141, 0.240 and 0.164, respectively. The LMO2 correlated gene numbers in normal, all tumor, Luminal A, Luminal B, Her2 and Basal subsets were 11160, 12162, 10865, 6605, 5205 and 5610, respectively (Supplementary Data Set Table 2.1).

### Bioinformatics analysis methods

For survival analysis, breast cancer samples in the whole sample set or in each subtype separately, were divided into LMO2-low and LMO2-high group based on the median, subsequently analysis was performed with IBM SPSS Statistics version 20.0. The KEGG, GO enrichment assay and GSEA were performed by R_ClusterProfiler package [25]. For GSEA, all LumA-Her2 differently LMO2-correlated genes were ranked by (*r*_(LumA)_-*r*_(Her2)_) value descendingly, and GSEA analysis was performed on this ranked genelist. The GSEA parameters were exponent = 1, nPerm = 1000, minGSSize = 10, maxGSSize=500, pvalueCutoff = 0.05, pAdjustMethod = “BH”. All images were drawn by R.

### Plasmid constructs and cell strain generation

LMO2 expression and control lentiviral vectors (with a luciferase fluorescent marker), LMO2-shRNA lentiviral vector (with an mCherry fluorescent marker), and the Lenti-Pac™ HIV Expression Packaging Kit were purchased from GeneCopoeia (Rockville, MD). The HEK293T packaging cells were used for lentiviral amplification according to GeneCopoeia’s instructions. ZR-75-1 and SKBR-3 cells were obtained from ATCC (University Boulevard, Manassas, VA) and regularly cultured in RPMI 1640 medium supplied with 10% FBS. Cells were infected by packaged LMO2 overexpression, control or LMO2-shRNA lentivirus for 24 hrs. Stable cell strains were selected in medium supplemented with 2 μg/mL puromycin three days after lentiviral infection and maintained in medium supplemented with 1 μg/mL puromycin till harvested.

### Gene expression microarray assay

Total RNA of each cell strain was isolated using Trizol reagent (Invitrogen, Austin, TX, USA). Double-strand cDNA (ds-cDNA) was synthesized from 5 μg of total RNA using an Invitrogen SuperScript ds-cDNA synthesis kit in the presence of 100 pmol oligo dT primers. ds-cDNA was cleaned and labeled in accordance with the NimbleGen Gene Expression Analysis protocol (NimbleGen Systems, Inc., USA). Hybridization was performed with the Human 12 × 135K Gene Expression Array manufactured by Roche NimbleGen. Slides were scanned at 5 μm/pixel resolution using an Axon GenePix 4000B scanner (Molecular Devices Corporation) piloted by GenePix Pro 6.0 software (Axon). Scanned images (TIFF format) were then imported into NimbleScan software (version 2.5) for grid alignment and expression data analysis. Expression data were normalized through quantile normalization and the Robust Multichip Average (RMA) algorithm included in the NimbleScan software. All original microarray data were available at GEO website (GEO accession number: GSE105020), the gene expression data matrix was also available in the Supplementary Data Set Table 7. Further data analysis was performed by R. The KEGG and GO enrichment assay were performed by R_ClusterProfiler package.

## SUPPLEMENTARY MATERIALS FIGURES AND TABLES














































