# Supplementary material for: A comprehensive function analysis of LMO2 in different breast cancer subtypes
Source: Oncotarget. 2017 Dec 21;9(10):8911–26. doi: 10.18632/oncotarget.23542 (PMC5823636; doi:10.18632/oncotarget.23542)
Supplement: Supplementary file 1 [file oncotarget-09-8911-s001.pdf]

A comprehensive function analysis of LMO2 in different breast cancer subtypes

SUPPLEMENTARY MATERIALS

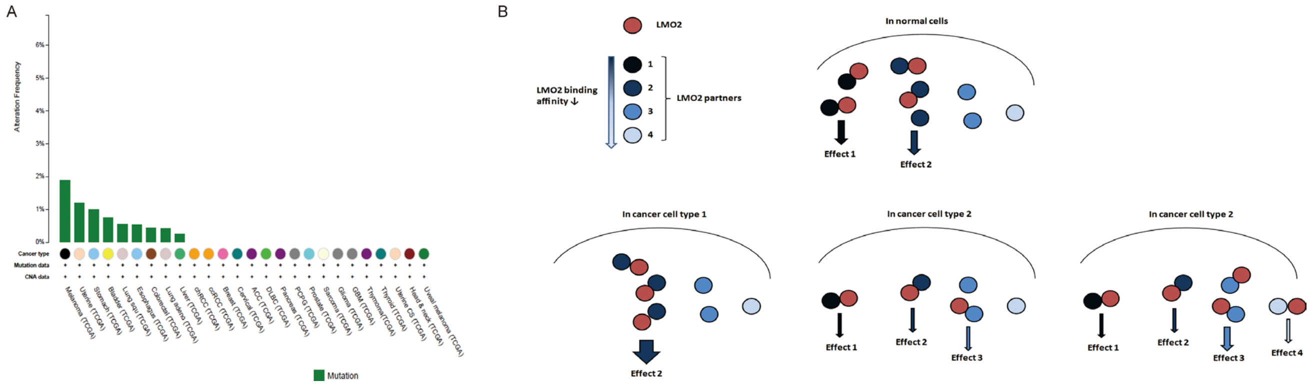

Supplementary Figure 1: Additional LMO2 expression and function features. (**A**) Mutation rate of LMO2 in pan-cancer data from TCGA dataset. No genebody mutation was found in breast cancers. (**B**) A sketch map showing the probably function pattern of LMO2. The competitive network may confer LMO2 functional complexity in different cellular circumstance.

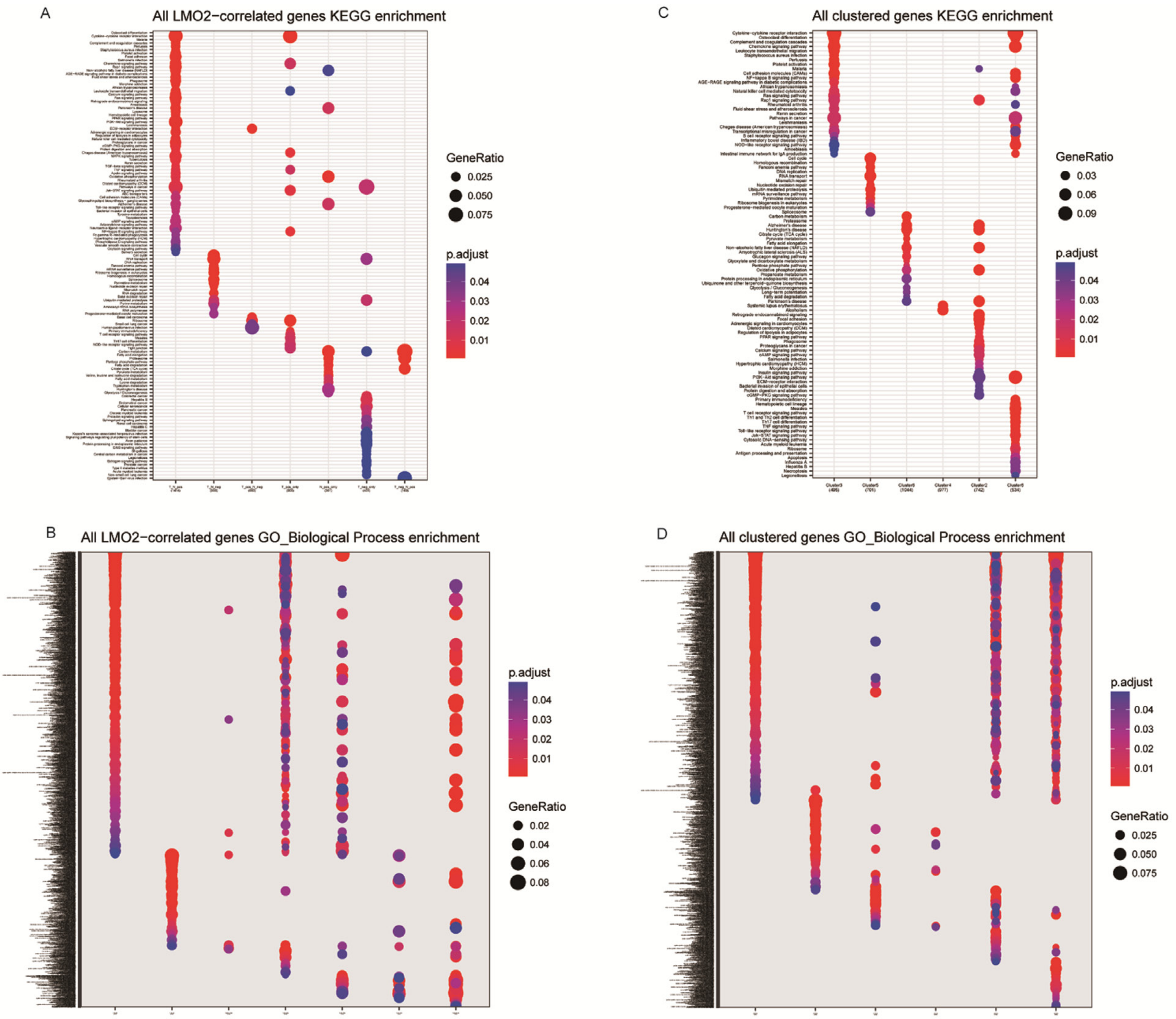

Supplementary Figure 2: Additional LMO2 function profiles in malignant and normal breast tissues. (**A**, **B**) Dot plots showing all terms of KEGG or additional GO-Biological Process (GO_BP) enrichment assay on each category of normal or tumor samples. Relative information was marked on the plots. (**C**, **D**) Dot plots showing all terms of KEGG or additional GO-Biological Process (GO_BP) enrichment assay on the 8 means clusters. Relative information was marked on the plots.

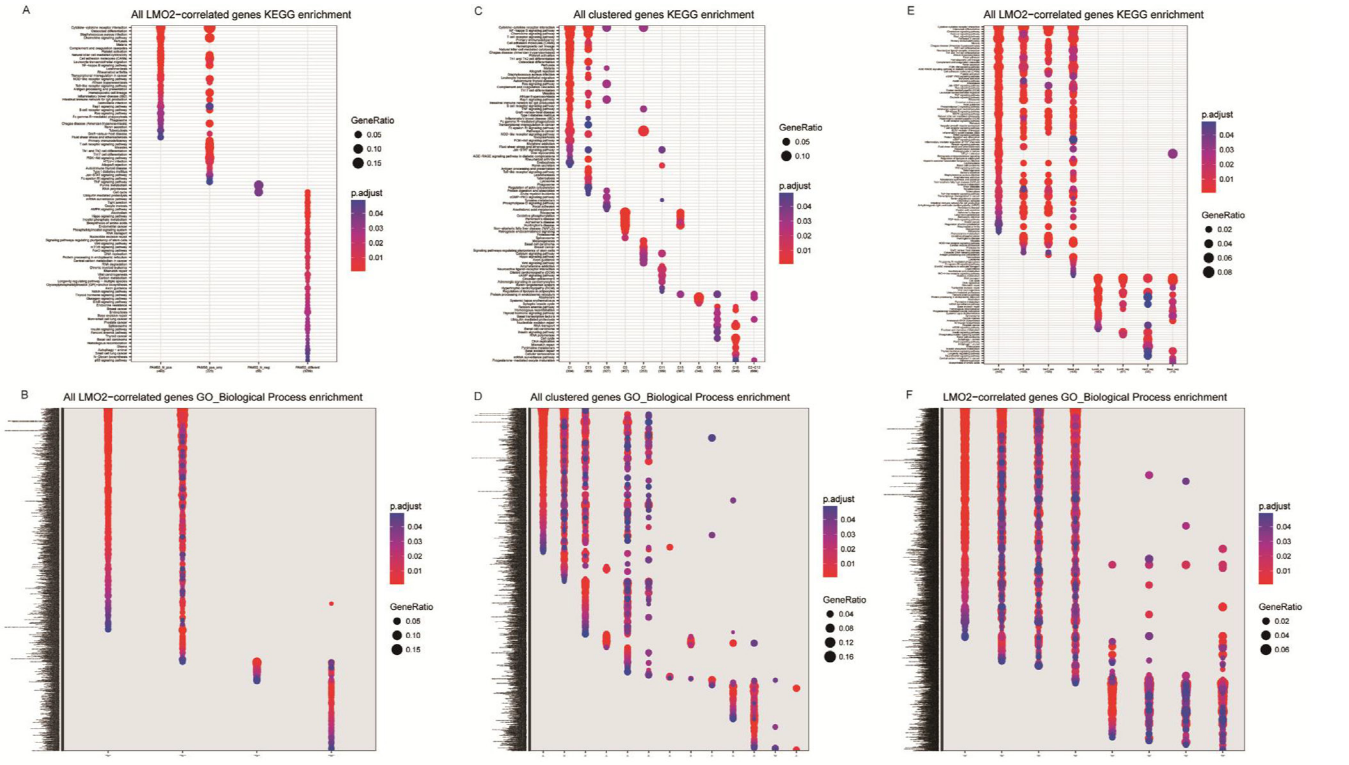

Supplementary Figure 3: Additional LMO2 function profiles in different PAM50 subtypes of breast cancer samples. (**A**, **B**) Dot plots showing all terms of KEGG or additional GO-Biological Process (GO_BP) enrichment assay on each category of PAM50 identical or different genesets. Relative information was marked on the plots. (**C**, **D**) Dot plots showing all terms of KEGG or additional GO-Biological Process (GO_BP) enrichment assay on the 18 means clusters. Relative information was marked on the plots. (**E**, **F**) Dot plots showing all terms of KEGG or additional GO-Biological Process (GO_BP) enrichment assay on each category of the 4 PAM50 subtype independently. Relative information was marked on the plots.

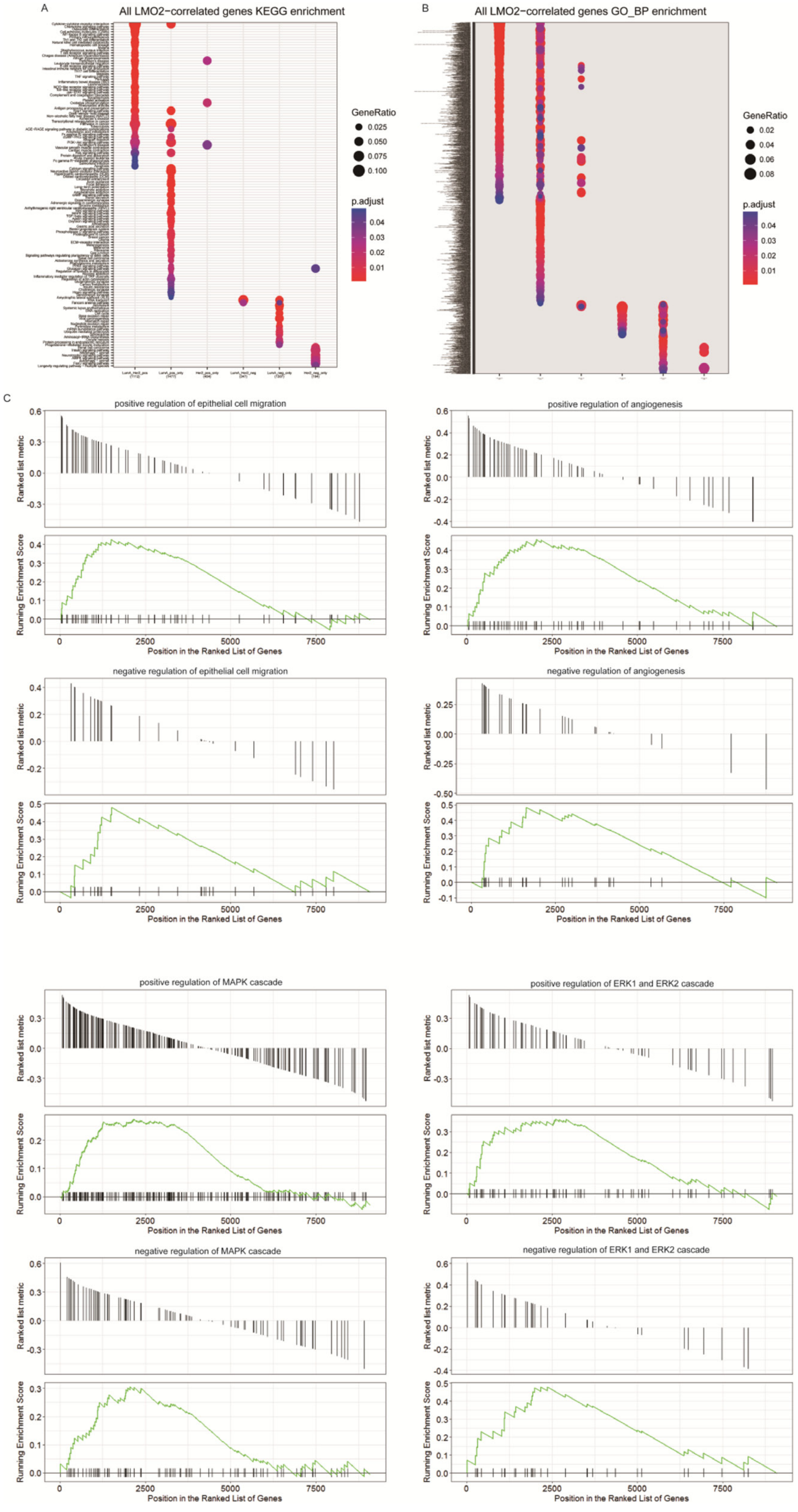

Supplementary Figure 4: Additional function profiles of LMO2 between Luminal A and Her2 subtype of breast cancers in TCGA dataset. (**A**, **B**) Dot plots showing all terms of KEGG or additional GO-Biological Process (GO_BP) enrichment assay on each geneset of the intersected or unique fractions of LMO2-correlated genes in Luminal A and Her2 subgroups. Relative information was marked on the plots. (**C**) Additional GSEA plots of selected, significantly enriched cancer-associated terms (q value < 0.05) but exhibiting dual directional regulation in the dataset of all LumA-Her2 differently LMO2-correlated genes (the 4 unique sections in A) ranked by r(LumA)-r(Her2) value. Other relative information was marked on the plots.

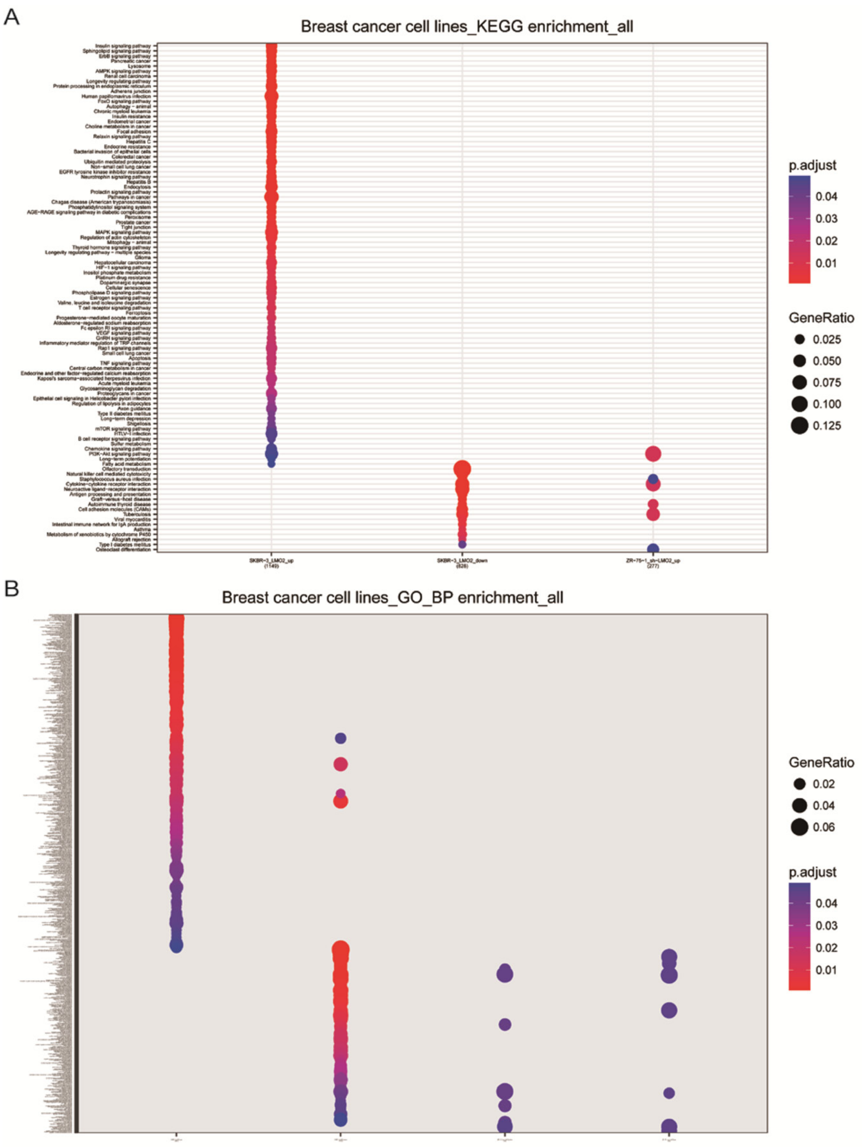

Supplementary Figure 5: Additional function profiles of LMO2 on Luminal A and Her2 like breast cancer cell lines. (**A**, **B**) Dot plots showing all terms of KEGG or additional GO-Biological Process (GO_BP) enrichment assay on each geneset in SKBR-3 and ZR-75-1 cells. Relative information was marked on the plots.

Supplementary Data Set Table 1: TCGA_breast cancer_survival_LMO2_data. See Supplementary_Data_Set_Table_1

Supplementary Data Set Table 2.1: LMO2_correlation_data_matrix. See Supplementary_Data_Set_Table_2.1

Supplementary Data Set Table 2.2: tn_kegg_all. See Supplementary_Data_Set_Table_2.2

Supplementary Data Set Table 2.3: tn_GO_BP_all. See Supplementary_Data_Set_Table_2.3

Supplementary Data Set Table 2.4: 8_Clustered_genes_KEGG_all. See Supplementary_Data_Set_Table_2.4

Supplementary Data Set Table 2.5: 8_Clustered_genes_GO_BP_all. See Supplementary_Data_Set_Table_2.5

Supplementary Data Set Table 3.1: PAM50_KEGG_all. See Supplementary_Data_Set_Table_3.1

Supplementary Data Set Table 3.2: PAM50_GO_BP_all. See Supplementary_Data_Set_Table 3.2

Supplementary Data Set Table 3.3: 18_Clustered_genes_KEGG_all. See Supplementary_Data_Set_Table 3.3

Supplementary Data Set Table 3.4: 18_Clustered_genes_GO_BP_all. See Supplementary_Data_Set_Table 3.4

Supplementary Data Set Table 3.5: PAM50_pn_KEGG_all. See Supplementary_Data_Set_Table 3.5

Supplementary Data Set Table 3.6: PAM50_pn_GO_BP_all. See Supplementary_Data_Set_Table 3.6

Supplementary Data Set Table 4.1: LumA_Her2_KEGG_all. See Supplementary_Data_Set_Table 4.1

Supplementary Data Set Table 4.2: LumA_Her2_GO_BP_all. See Supplementary_Data_Set_Table_4.2

Supplementary Data Set Table 4.3: LumA_Her2_GSEA_KEGG_all. See Supplementary_Data_Set_Table_4.3

Supplementary Data Set Table 4.4: LumA_Her2_GSEA_GO_BP_all. See Supplementary_Data_Set_Table_4.4

Supplementary Data Set Table 5.1: Breast_cancer_cell_lines_KEGG_result. See Supplementary_Data_Set_Table_5.1

Supplementary Data Set Table 5.2: Breast_cancer_cell_lines_GO_BP_result. See Supplementary_Data_Set_Table_5.2

Supplementary Data Set Table 5.3: Subsets of breast cancer cells_KEGG_result. See Supplementary_Data_Set_Table 5.3

Supplementary Data Set Table 5.4: Subsets of breast cancer cells_GO_BP_result. See Supplementary_Data_Set_Table 5.4

Supplementary Data Set Table 6: TCGA_LMO2_clinical_information. See Supplementary_Data_Set_Table 6

Supplementary Data Set Table 7: Breast cancer cell lines_array_data. See Supplementary_Data_Set_Table 7
